# Low Sucrose, Omega-3 Enriched Diet Has Region-Specific Effects on Neuroinflammation and Synaptic Function Markers in a Mouse Model of Doxorubicin-Based Chemotherapy

**DOI:** 10.3390/nu10122004

**Published:** 2018-12-18

**Authors:** Tonya S. Orchard, Monica M. Gaudier-Diaz, Panchita Phuwamongkolwiwat-Chu, Rebecca Andridge, Maryam B. Lustberg, Joshua Bomser, Rachel M. Cole, Martha A. Belury, A. Courtney DeVries

**Affiliations:** 1 Department of Human Sciences, Human Nutrition Program, The Ohio State University, Columbus, OH 43210, USA; phuwamongkolwiwat.1@osu.edu (P.P.-C.); bomser.1@osu.edu (J.B.); cole.311@osu.edu (R.M.C.); belury.1@osu.ed (M.A.B.); 2 Department of Psychology and Neuroscience, University of North Carolina, Chapel Hill, NC 27707, USA; gaudier@email.unc.edu; 3 Division of Biostatistics, The Ohio State University, Columbus, OH 43210, USA; andridge.1@osu.edu; 4 Division of Medical Oncology, The Ohio State University, Columbus, OH 43210, USA; maryam.lustberg@osumc.edu; 5 Department of Neuroscience, West Virginia University, Morgantown, WV 26506, USA; Courtney.devries@hsc.wvu.edu

**Keywords:** chemotherapy, doxorubicin, neuroinflammation, omega-3 fatty acids, sucrose

## Abstract

Chemotherapeutic agents such as doxorubicin may negatively affect long-term brain functioning in cancer survivors; neuroinflammation may play a causal role. Dietary approaches that reduce inflammation, such as lowering sucrose and increasing eicosapentaenoic acid plus docosahexaenoic acid (EPA + DHA), may attenuate chemotherapy-induced neuroinflammation and synaptic damage, thereby improving quality of life. Ovariectomized, C57BL/6 mice were assigned to a chemotherapy (9 mg/kg doxorubicin + 90 mg/kg cyclophosphamide) or vehicle two-injection regimen, with injections two and four weeks after starting diets. In Study 1, mice received low sucrose diets with EPA + DHA or No EPA + DHA for four to six weeks; tissues were collected four, seven, or 14 days after the second injection. Compared to vehicle, chemotherapy increased pro-inflammatory cytokine IL-1β at day seven in the cortex and hippocampus, and reduced gene expression of synaptic marker *Shank 3* at all timepoints in cortex, while EPA + DHA increased expression of *Shank 3*. In Study 2, high or low sucrose/EPA + DHA or No EPA + DHA diets were fed for five weeks; tissues were collected ten days after the second injection. Among chemotherapy-treated mice, brain DHA was higher with low sucrose feeding. Furthermore, low sucrose increased gene expression of *Shank 1*, while EPA + DHA increased expression of *Shank 3* and reduced protein concentrations of pro-inflammatory markers IL-5, IL-6 and KC/GRO in the cortex, but not the hippocampus. Low sucrose, EPA + DHA diets may attenuate neuroinflammation and synaptic damage induced by doxorubicin-based chemotherapy in specific brain regions.

## 1. Introduction

Chemotherapy treatment for breast cancer has contributed to improved survival rates for millions of women [1]. However, chemotherapeutic agents, such as doxorubicin, which are frequently used to treat breast cancer and other solid tumors, have several detrimental side effects, including toxicities that affect normal functioning of the brain [2]. Studies report cognitive side effects in up to 75% of individuals treated with chemotherapy for cancer [1]; indeed, cognitive deficits negatively impact quality of life in nearly 30% of breast cancer survivors [3,4] by interfering with activities of daily living such as reading, working, and driving [5]. These cognitive deficits may persist for up to 15 years post-treatment [6], affecting verbal fluency, working memory and processing speed [7]. Common side effects of chemotherapy not only include cognitive deficits, but other behavioral toxicities such as sleep disturbances and associated fatigue [8,9], affect disorders [10] and neuropathy [8]. Although the mechanisms underlying chemotherapy-induced behavioral toxicities have not been conclusively established, research suggests that neuroinflammation, oxidative stress and their effects on neuronal structure and function may play a key role [8,9,11,12,13,14].

Dietary approaches that reduce inflammation and oxidative stress are potential strategies for alleviating negative cognitive outcomes associated with chemotherapy. The omega-3 polyunsaturated fatty acids (n-3 PUFAs), eicosapentaenoic acid (EPA) and docosahexaenoic acid (DHA), found primarily in fish, have well-established anti-inflammatory properties [15]. Recent animal research found that administration of EPA and DHA concurrently with doxorubicin chemotherapy reduced neuroinflammation, oxidative stress, and neural apoptosis, and prevented depressive-like symptoms [16]. In lung cancer patients undergoing chemotherapy, EPA and DHA supplementation reduced plasma markers of inflammation and oxidative stress compared to placebo [17]. In breast cancer survivors, dietary EPA and DHA were associated with lower inflammation after 24 weeks of aromatase-inhibitor treatment [18]. Incorporation of DHA in particular, into plasma membranes, was associated with improved overall survival in women with advanced metastatic breast cancer during doxorubicin-based chemotherapy [19].

In contrast to the potential benefits of EPA and DHA on inflammation and behavioral outcomes, high amounts of dietary added sugars may act independently to promote cognitive deficits [20] and systemic inflammation [21], or interact with n-3 PUFAs to diminish their anti-inflammatory actions [22] and cause changes in brain n-3 PUFA metabolism [23]. Evidence from a rodent model of metabolic syndrome suggests that a diet rich in n-3 PUFAs is effective at alleviating some of the deleterious outcomes of high added sugar consumption on memory [20]. Whether chemotherapy-induced physiological changes in the brain are amplified by high sugar intake, or if dietary enrichment with n-3 PUFAs can attenuate neuroinflammation, oxidative stress, and structural changes in the context of a high sugar diet, has not been previously studied. To address this translationally important research gap, we undertook several experiments to investigate these interactions and underlying mechanisms using a postmenopausal mouse model of chemotherapy to mirror treatment commonly administered to breast cancer patients. We hypothesized that a low sucrose, high EPA + DHA diet would protect the brain from the neuroinflammatory and synaptic damage associated with doxorubicin-based chemotherapy.

## 2. Materials and Methods

### 2.1. Animals and Treatments

These studies were conducted in accordance with the National Institute of Health Guide for the Care and Use of Laboratory Animals and under protocols approved by the Ohio State Institutional Animal Care and Use Committee. The mice were randomly assigned to groups and all data were collected by individuals who were uninformed of experimental assignment.

Adult (~8 weeks old) C57BL/6 female mice were purchased from Charles River Laboratories (Wilmington, MA, USA). Upon arrival, mice were housed in an Association for Assessment and Accreditation of Laboratory Animal Care-approved vivarium and acclimated to the facility at a 14:10 light/dark cycle. All animals were provided ad libitum access to standard chow and water. The mice were ovariectomized using a sterile surgical technique; buprenorphine was provided for analgesia. One week later, they were weighed, transferred to individual cages, and started on semi-purified diets.

Four modified AIN-76A diets were produced by Research Diets Inc. (Newark, NJ, USA): a 2% EPA + DHA/Low sucrose diet containing 2% of kcal as EPA + DHA and 9% of kcal as sucrose, a No EPA + DHA/Low sucrose diet, a No EPA + DHA/High sucrose diet containing 47% kcal as sucrose, and a 2% EPA + DHA/High sucrose. The diets contained the same total macronutrients, kilocalories, fiber, vitamins, minerals, and other antioxidants (Supplementary Table S1). Fatty acid composition of the diets was measured by gas chromatography (Table S2). Diets were stored under refrigeration (4 °C) in the dark to prevent degradation, and were replaced every 3–4 days and food intake was recorded until completion of each study.

Mice were randomly assigned to the chemotherapy or saline treatment group at the beginning of each study. They received a total of two tail vein injections of either a cocktail of 9 mg/kg doxorubicin plus 90 mg/kg cyclophosphamide or sterile isotonic saline vehicle, two and four weeks after beginning diets. This chemotherapy dose was calculated to be the mouse equivalent of 50% of the typical human dose based on a body surface area equation [24]. Body weight was recorded prior to each injection.

### 2.2. Experimental Design

Study 1: Omega-3 and Chemotherapy Timecourse Experiment. One week after ovariectomy, mice (*n* = 60/diet) were randomized to No EPA + DHA/Low sucrose or 2% EPA + DHA/Low sucrose diets and the injection regimen described above. Mice were sacrificed and tissues were collected 4, 7 or 14 days after the second injection to determine changes in markers of central and peripheral inflammation.

Study 2: Sucrose, Omega-3, Chemotherapy and Brain Physiology Experiment. One week after ovariectomy, mice (*n* = 26/diet) were randomized to one of four diets (2% EPA + DHA/Low sucrose diet, No EPA + DHA/Low sucrose diet, No EPA + DHA/High sucrose diet, and 2% EPA + DHA/High sucrose diet). As in Study 1, two weeks after beginning diets, mice received the first injection of chemotherapy or vehicle, followed by a second injection two weeks later. In this study, tissues were collected at only one time point, ten days after the second injection.

### 2.3. Blood and Tissue Collection

Following blood collection from the submandibular vein, mice were injected with euthasol (270 mg/kg). Deeply anesthetized mice were perfused transcardially with 0.1 M PBS; then, brain was dissected and one hemisphere was flash frozen and stored at −80 °C for fatty acid and oxidative stress analysis, while the other hemisphere was stored in RNA-Later Reagent (Qiagen, Germantown, MD, USA) for protein and/or gene expression analysis.

### 2.4. RNA Preparation and Quantitative Polymerase Chain Reaction

Messenger RNA (mRNA) and protein were extracted from the hippocampus and cortex using TriZol reagent (Life Technologies, Thermo Fischer Scientific Inc., Waltham, MA, USA) and following the manufacturer’s instructions. Following the chloroform incubation and centrifugation RNA was extracted from the aqueous phase, while protein was extracted from the organic phase. mRNA quality and quantity were determined using a spectrophotometer (NanoDrop, Thermo Fisher Scientific Inc.). Then, cDNA was synthesized using M-MLV reverse transcription and diluted 1:10 for subsequent qPCR. mRNA concentrations of pro-inflammatory markers such as cytokines and nuclear factor κ-β (*Nfκ-β*), and synaptic proteins such as SH3 and multiple ankyrin repeat domains protein 1 (*Shank 1*) and 3 (*Shank 3*), were determined using a spectrophotometer (NanoDrop, Thermo Fischer Scientific Inc.). The qPCR reaction was performed using endogenous control eukaryotic 18S, probes from Applied Biosystems (see Table S3 for the list of primers), and Taqman Advanced Universal Master Mix (all from Life Technologies), on an ABI 7000 Sequencing Detection System. Relative gene expression was quantified by normalizing the target gene with endogenous control 18S.

### 2.5. Cytokine and Chemokine Protein Assays

Serum, hippocampal and cortical samples were analyzed for protein levels of tumor necrosis factor-α (TNF-α), interferon-γ (IFN-γ), interleukin-10 (IL-10), IL-12p70, IL-1β, IL-2, IL-4, IL-5, IL-6, and keratinocyte chemoattractant/human growth-regulated oncogene (KC/GRO, also known as CXCL1) using a multiplex assay (V-Plex proinflammatory panel 1, Meso-Scale Discovery, Rockville, MD, USA).

### 2.6. Fatty Acid Analysis

Total lipids from one brain hemisphere were extracted [25] and methylated [26], and analysis of fatty acid methyl esters was completed by gas chromatography as previously described [27]. Retention times were compared to authentic standards for fatty acid methyl esters (Nu-Check Prep Inc., Elysian, MN, USA and Matreya, Inc., Pleasant Gap, PA, USA) and fatty acids were reported as percent of total identified.

### 2.7. Lipid Peroxidation and Oxidative Stress Assays

Total protein was extracted from brain tissue lysate using a Pierce BCA Protein Assay Kit (Fisher Scientific, Waltham, MA, USA) and quantified by spectrophotometry. Following protein extraction, brain lysates containing PBS and protease inhibitor were derivatized for carbonyl groups using the Oxyblot Kit (EMD Millipore, Billerica, MA, USA) according to the manufacturer’s instruction. Proteins were loaded on a 12% SDS-PAGE and electrotransferred to a nitrocellulose membrane (Bio-Rad Laboratories, Hercules, CA, USA). Membranes were incubated in blocking buffer (1% BSA in 1× PBS-T) at room temperature for 1 h followed by incubation overnight at 4 °C with the primary Rabbit Anti-DNP antibody, then incubation with secondary antibodies (Goat Anti-Rabbit IgG; HRP-conjugated) at room temperature for 1 h. Bands were detected by chemiluminescence using Odyssey^®^ Fc Imaging System (LI-COR Biosciences, Lincoln, NE, USA).

As a marker of lipid peroxidation, levels of 4-hydroxynonenal–histidine (4-HNE) protein adducts in brain were assayed using the Oxiselect HNE–His Adduct ELISA Kit (EMD Millipore, Billerica, MA, USA) and normalized to protein levels. Oxidative stress was assayed in brain lysates derivatized for carbonyl groups using the Oxyblot Kit (EMD Millipore, Billerica, MA, USA). Superoxide dismutase (SOD) was measured using an activity assay (Cell Biolabs Inc, San Diego, CA, USA). Brain samples were prepared by homogenizing in lysis buffer (10 mM Trish, 150 mM NaCl, 0.1 mM EDTA) and centrifuging at 12,000× *g* for 10 min. The supernatant was extracted and plated with standards according to the kit instructions. The absorbance was read at 490 nm and data is expressed as percent inhibition of superoxide anions.

### 2.8. Statistical Analysis

Grubbs test was used to detect outliers within groups; data values corresponding to a Z-score > 2 were removed prior to completing statistical analysis and graphing. For outcome measures where a large fraction of samples (>25%) were below the detection limit, no analysis was completed. Gene expression, protein, fatty acids, peroxidation, oxidative stress, food intake, and behavior data were analyzed using three-way analysis of variance (ANOVA). Factors for study 1 were injection type (chemo, vehicle), omega-3 (2% EPA + DHA, No EPA + DHA), and day (4, 7, 14 days post-injection). Factors for study 2 were injection type (chemo, vehicle), sucrose (high, low), and omega-3 (2% EPA + DHA, No EPA + DHA). Repeated measures ANOVA was used to evaluate body weight changes. When the overall ANOVA F-test was significant (*p* < 0.05), significant interactions and lower order terms were examined and graphed, and pairwise comparisons were made by comparing the groups within a single variable (e.g., vehicle versus chemo and 2% EPA + DHA versus No EPA + DHA). Results are reported as group means averaged over any factors not involved in the interaction term or main effect. Statistical analysis was completed with SAS software version 9.4 (Cary, NC, USA). In graphs, data are presented as mean ± standard error of the mean (SEM).

## 3. Results

### 3.1. Effects of Omega-3 Fatty Acids and Chemotherapy on Food Intake and Body Weight

In study 1, there was a significant effect of diet on cumulative food intake (*p* < 0.001) such that mice in the No EPA + DHA groups (both chemotherapy and vehicle) averaged 6% greater food intake than mice in the 2% EPA + DHA/chemotherapy group (all *p* < 0.05) at end of the study (Table S4). There were no significant differences in body weight between diet groups at baseline, day 14 or day 28 (all *p* > 0.05). However, there was a significant injection by day effect on body weight (*p* < 0.0001); specifically, mice in the chemotherapy groups lost a mean of 0.72 ± 1.03 g of body weight from first injection to second injection, while mice in vehicle groups gained a mean of 0.30 ± 1.57 g. (Table S4).

### 3.2. Effects of Omega-3 Fatty Acids and Chemotherapy on Serum Protein Concentrations of Cytokines and Chemokines over Time

**Figure 1:**
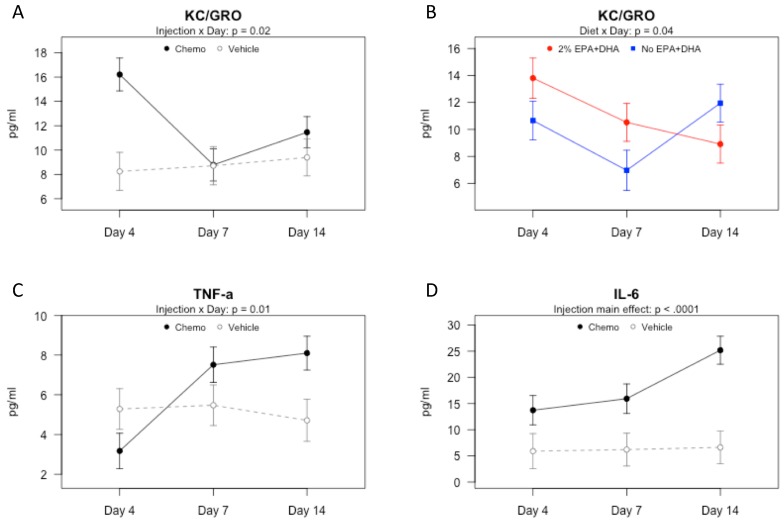
Serum cytokines and chemokines in mice receiving 2% EPA + DHA or No EPA + DHA diets and vehicle or chemotherapy 4, 7, or 14 days (D4, D7, D14) after second injection (Study 1). Proteins concentrations measured by multiplex ELISA assay. Data (means ± SEM, *n* = 8–12 mice per group) were analyzed by 3-way ANOVA. (**A**) Injection by day interaction effect on keratinocyte chemoattractant/human growth-regulated oncogene (KC/GRO). (**B**) Diet by day interaction effect on KC/GRO. (**C**) Injection by day interaction effect on tumor necrosis factor α (TNF-α). (**D**) Main effect of injection on serum Interleukin 6 (IL-6).

Protein concentrations for IL-1β, IL-5, IL-6, KC/GRO and TNF-α were measured; IFN-γ, IL-10, IL-12p70, IL-2 and IL-4 were not analyzed because >25% of samples were below the detection limits in study 1. We observed differences in the cytokine pattern between chemotherapy and vehicle groups over time, as has been reported in other models of inflammation [28,29]. We observed an initial rise in KC/GRO, also known as CXCL1, a chemokine responsible for recruitment of peripheral mononuclear cells to the site of inflammation [30]. There was an injection by day interaction (Figure 1A; *p* < 0.05), such that the chemotherapy group had higher mean KC/GRO than the vehicle group 4 days after second injection only (*p* < 0.001). There was also a significant n-3 PUFAs by day interaction on KC/GRO (Figure 1B; *p* < 0.05) such that in the EPA + DHA group, KC/GRO concentration decreased over time, whereas in the No EPA + DHA group it decreased from 4 to 7 days post-chemotherapy and then increased from 7 to 14 days. Serum TNF-α varied significantly among groups (Figure 1C; *p* < 0.01). There was a significant injection by tissue collection day interaction (*p* < 0.05) on serum TNF-α; specifically, TNF-α concentration increased over time in the chemotherapy group. By day 14, the mean for the chemotherapy group was significantly higher than the vehicle group (*p* < 0.05). IL-6 was significantly higher among the chemotherapy than vehicle groups at all time points (Figure 1D; *p* < 0.0001), suggesting a sustained effect of chemotherapy on this cytokine, as has been noted in breast cancer patients undergoing doxorubicin plus cyclophosphamide treatment [31]. Altogether, these findings suggest that chemotherapy leads to systemic inflammation, an effect that can be partly moderated by n-3 PUFAs.

### 3.3. Effects of Omega-3 Fatty Acids and Chemotherapy on Gene Expression of Cytokines, Chemokines, and Shank in the Brain over Time

**Figure 2:**
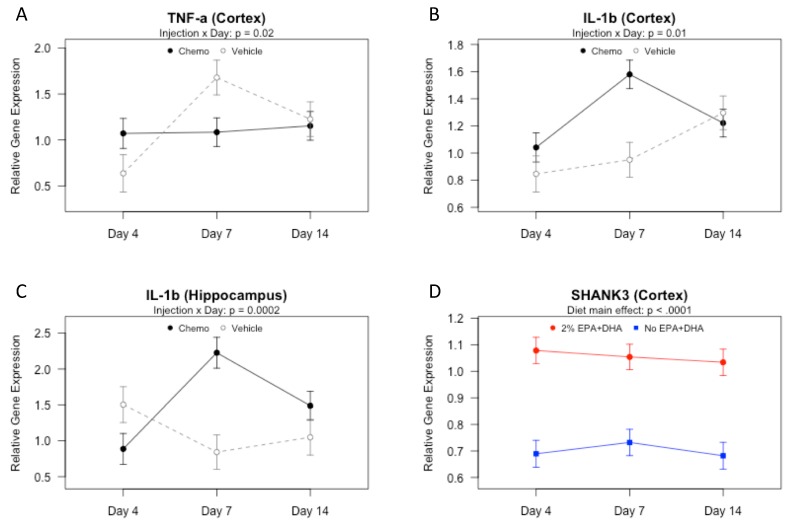
Cortical and hippocampal gene expression of cytokines and Shank 3 in mice receiving 2% EPA + DHA or No EPA + DHA diets and vehicle or chemotherapy 4, 7, or 14 days (D4, D7, D14) after second injection (Study 1). mRNA measured by q-PCR. Data (means ± SEM, *n* = 8–12 mice per group) were analyzed by 3-way ANOVA. (**A**) Injection by day interaction effect on TNF-a in cortex. (**B**) Injection by day interaction effect on IL-1b in cortex. (**C**) Injection by day interaction effect on IL-1b in hippocampus. (**D**) Main effect of diet on Shank 3.

In study 1, cortex gene expression of *Tnf-α* (*p* = 0.05) and *Il-1β* (*p* < 0.01) varied significantly among the groups. No significant effects of EPA + DHA diets were found. However, there was an injection by day interaction on *Tnf-α* (Figure 2A; *p* < 0.05): although *Tnf-α* remained relatively consistent over time in the chemotherapy group, *Tnf-α* was elevated in the vehicle group relative to the chemotherapy group 7 days post-injection (*p* < 0.05). This might result from a negative feedback loop in cytokine signaling [32]. Cortical *Il-1β* gene expression also exhibited an injection by day interaction (Figure 2B; *p* < 0.05). At 7 days post-injection, the chemotherapy group had significantly higher *Il-1β* expression than the vehicle treated group (*p* < 0.001). There were no significant group differences in expression of *Il-6* or *Nfκ-β* (*p* > 0.05 for both).

There were no significant group differences in hippocampal gene expression of *Tnf-α*, *Il-6*, or *Nfκ-β* (*p* > 0.05 for all). However, results differed by group for *Il-1β* (*p* < 0.01); there was an interaction between the injection and day on hippocampal *Il-1β* (Figure 2C; *p* < 0.001). At 7 days post-injection, the chemotherapy mice expressed significantly higher *Il-1β* than the vehicle treated mice (*p* < 0.0001). These data suggest that, of the time points measured, 7 days after the second chemotherapy injection was the most neuroinflammatory, and neuroinflammation was mediated primarily through IL-1β, as peripheral TNF-α was elevated at this time point (Figure 1C), but chemotherapy did not increase gene expression of TNF-α in the brain at that time (Figure 2A).

Gene expression of *Shank 3*, a postsynaptic density protein that participates in synapse formation and dendritic spine maturation [33], was examined in the hippocampus and cortex. Hippocampal *Shank 3* gene expression remained constant throughout the experimental groups and time points investigated (data not shown; *p* > 0.05). However, in the cortex, the protective effect of EPA and DHA was demonstrated as *Shank 3* gene expression was significantly higher for 2% EPA + DHA groups, irrespective of injection treatment or day of tissue collection (Figure 2D; *p* < 0.0001). These data suggest that in the presence or absence of a chemotherapy-induced inflammatory environment, n-3 administration may facilitate synapse formation in the cortex.

### 3.4. Effects of Sucrose, Omega-3 Fatty Acids, and Chemotherapy on Food Intake and Body Weight

In Study 2, food consumption was significantly different between groups (See Table S5). Total food consumption was 4% higher for vehicle relative to chemotherapy groups (*p* < 0.05) and No EPA + DHA versus 2% EPA + DHA (*p* < 0.01), and 5% higher for high sucrose versus low sucrose groups (*p* < 0.01). There was a significant effect of sucrose on body weight (Table S5); averaging across injection groups, n-3 PUFA groups and time, mice in high sucrose versus low sucrose groups had greater body (*p* < 0.05). There also was a significant injection by day effect on weight (*p* < 0.0001); the chemotherapy group lost an average of 0.67 g and the vehicle group gained an average of 0.70 g between the first and second injection (*p* < 0.001; Table S5).

### 3.5. Effects of Sucrose, Omega-3 Fatty Acids, and Chemotherapy on Brain Fatty Acids

**Figure 3:**
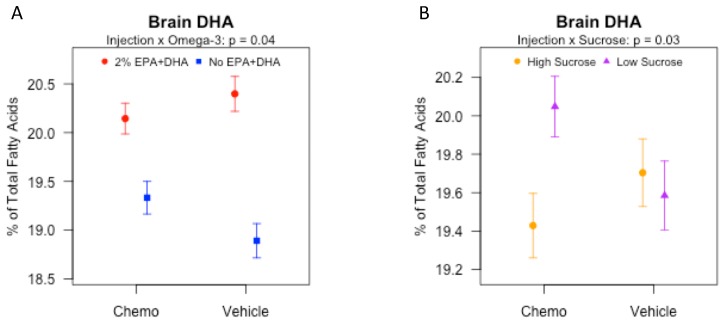
Effects of injection, omega-3, and dietary sucrose on brain docosahexaenoic acid (C22:6n-3) in mice receiving various combinations of omega-3 and sucrose diets and two vehicle or chemotherapy injections (Study 2). Brain fatty acids (FA) were measured by gas chromatography and reported as % of total FAs. Data (means ± SEM; *n* = 11–14/group) were analyzed by 3-way ANOVA. (**A**) Injection by omega-3 interaction effect. (**B**) Injection by dietary sucrose interaction effect.

In study 2, analysis of brain fatty acids by gas chromatography revealed significant differences in numerous n-3 and n-6 fatty acids (Table S6). As expected, the brain n-3 PUFA, EPA (C20:5n-3) and docosapentaenoic acid n-3 (C22:5n-3), were significantly higher (*p* < 0.0001 for both) and the n-6 fatty acids, linoleic acid (C18:2n-6), arachidonic acid (C20:4n-6) and docosatetraenoic acid (C22:4n-6) were significantly lower (all *p* < 0.001) in the 2% EPA + DHA compared to No EPA + DHA diet groups. Additionally, there was a significant main effect of sucrose on linoleic acid (*p* < 0.05) such that brain linoleic acid was significantly higher in low sucrose versus high sucrose groups. There was a significant interaction of injection by n-3 diet group (*p* < 0.05, Figure 3A), such that brain DHA (C22:6n-3) was significantly higher in mice fed 2% versus No EPA + DHA diets in both the chemotherapy (*p* < 0.01) and vehicle (*p* < 0.0001) groups, but the difference was significantly larger for vehicle treated groups (*p* < 0.05). Interestingly, there was a significant injection by sucrose interaction on DHA (Figure 3B; *p* < 0.05) such that brain DHA was significantly higher in the mice fed low sucrose versus high sucrose diets in the chemotherapy group (*p* < 0.01) but not in the vehicle group (*p* > 0.05).

### 3.6. Effects of Sucrose, Omega-3 Fatty Acids, and Chemotherapy on Serum Protein Concentrations of Cytokines and Chemokines

**Figure 4:**
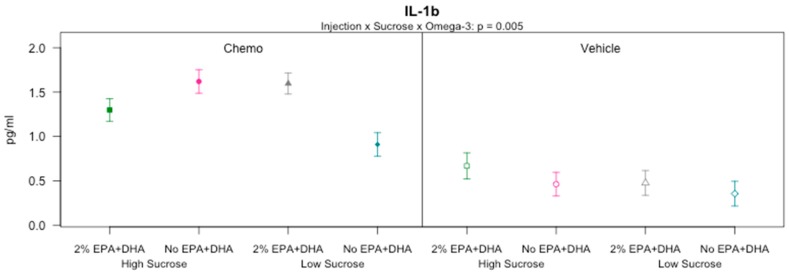
Serum IL-1β by group in mice receiving various combinations of omega-3 and sucrose diets and two vehicle or chemotherapy injections (Study 2). Proteins measured by multiplex ELISA assay. Data (means ± SEM; *n* = 11–14/group) were analyzed by 3-way ANOVA. Three-way interaction effect of injection by sucrose by omega-3 on IL-1b.

In the serum, protein concentrations of TNF-α, IL-1β, IL-6, IFN-γ, IL-5, KC/GRO, IL-10, and IL-2 were analyzed (Table 1); IL-12p70 and IL-4 were not analyzed because >25% of samples were below the detection limits in study 2 (data not shown). Serum concentrations of IL-10, IL-2 and KC/GRO did not vary across experimental groups (*p* > 0.05 for all). TNF-α (*p* ≤ 0.01), IL-6 (*p* < 0.0001), IFN-γ (*p* ≤ 0.01), and IL-5 (*p* < 0.0001) varied among the experimental groups, displaying an injection mediated main effect where the chemotherapy mice exhibited increased cytokine expression relative to the vehicle mice. IL-1β also varied significantly between groups (*p* < 0.0001), with a significant three-way interaction between chemotherapy, sucrose and EPA + DHA (Figure 4; *p* < 0.01). Among the chemotherapy groups, the mice receiving a low sucrose and No EPA + DHA diet expressed significantly lower IL-1β than all other groups (*p* < 0.05 for all comparisons). Among the vehicle treated mice, there were no significant differences (*p* > 0.05 for all comparisons). Taken together, these data demonstrate chemotherapy-induced inflammation in the periphery, which can be partially attenuated by low sucrose feeding in the absence of n-3 enrichment.

### 3.7. Effects of Sucrose, Omega-3 Fatty Acids, and Chemotherapy on Protein Concentrations of Cytokines and Chemokines in the Brain

Protein concentrations of IFN-γ, IL-10, IL-12p70, IL-1β, IL-2, IL-4, IL-5, IL-6, KC/GRO and TNF-α were quantified in the hippocampus and cortex in study 2. In the hippocampus data, >25% of samples were below detection limits for IFN-γ, IL-12p70, IL-1β, IL-2, IL-4, KC/GRO, and TNF-α, so no statistical analysis was completed. IL-10 concentration in the hippocampus did not vary among groups ((Table 1); *p* > 0.05). Hippocampal concentrations of IL-5 and IL-6 displayed similar patterns of variation among groups (*p* < 0.05 for both). The chemotherapy group had elevated IL-5 and IL-6 relative to the vehicle group (*p* < 0.05, main effect of injection), and the 2% EPA + DHA group had elevated IL-5 and IL-6 relative to the No EPA + DHA group (*p* < 0.05, main effect of omega-3 for both). Contrary to our hypothesis, these findings suggest that the chemotherapy-induced hippocampal increase of pro-inflammatory cytokines IL-5 and IL-6 can be exacerbated by n-3 PUFA.

In the cortex, analysis for all cytokines was completed (Table 1). Cortical IL-5, IL-6, and KC/GRO were the only cytokines that varied among groups (*p* < 0.05 for all). All three cytokines displayed a main effect of EPA + DHA (*p* < 0.01 for all); in contrast to effects of EPA + DHA in the hippocampus, the 2% EPA + DHA group had lower IL-5, IL-6 and KC/GRO concentrations relative to the No EPA + DHA group, demonstrating that EPA + DHA reduces inflammation in the cortex. Discrepancy between the hippocampal and cortical findings supports brain region specific effects of both the diet and chemotherapy treatment.

### 3.8. Effects of Sucrose, Omega-3 Fatty Acids, and Chemotherapy on Gene Expression of Inflammatory and Synaptic Markers in the Brain

**Figure 5:**
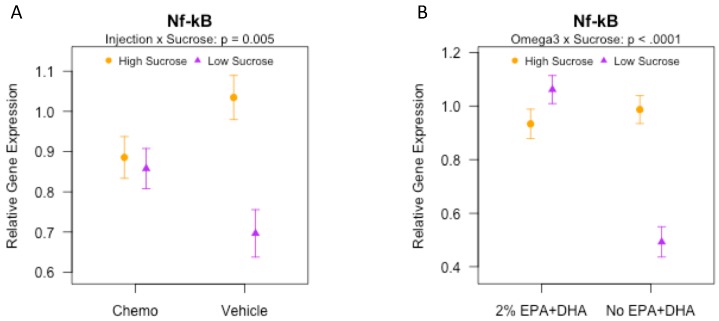
Hippocampal gene expression of *Nf-κβ* in mice receiving various combinations of omega-3 and sucrose diets and two vehicle or chemotherapy injections (Study 2). mRNA measured by q-PCR. Data (means ± SEM, *n* = 11–14/group) were analyzed by 3-way ANOVA. (**A**) Significant injection by sucrose interaction effect on *Nf-κβ*. (**B**) Significant sucrose by omega-3 interaction effect on *Nf-κβ*.

Although *Nf-κβ* did not vary appreciably in the chemotherapy treated mice (Figure 5A), among mice fed the low sucrose diets, *Nf-κβ* was elevated in chemotherapy relative to vehicle treated mice (*p* < 0.05); in contrast, among the high sucrose diets, the difference trended in the opposite direction (*p* = 0.052). Mice fed No EPA + DHA/High sucrose exhibited increased *Nf-κβ* relative to mice fed a No EPA + DHA/Low sucrose (Figure 5B; *p* < 0.0001). Among the 2% EPA + DHA diet groups, sucrose did not influence the hippocampal expression of *Nf-κβ* (*p* > 0.05). In the cortex, gene expression of *Tnf-α*, *Il-1β*, *Il-6,* and *Nf-κβ* did not vary among the experimental groups (*p* > 0.05 for all; Table 2). These changes in *Nf-κβ* suggest that low sucrose consumption can attenuate neuroinflammation in the hippocampus in the absence of chemotherapy and dietary EPA + DHA.

**Figure 6:**
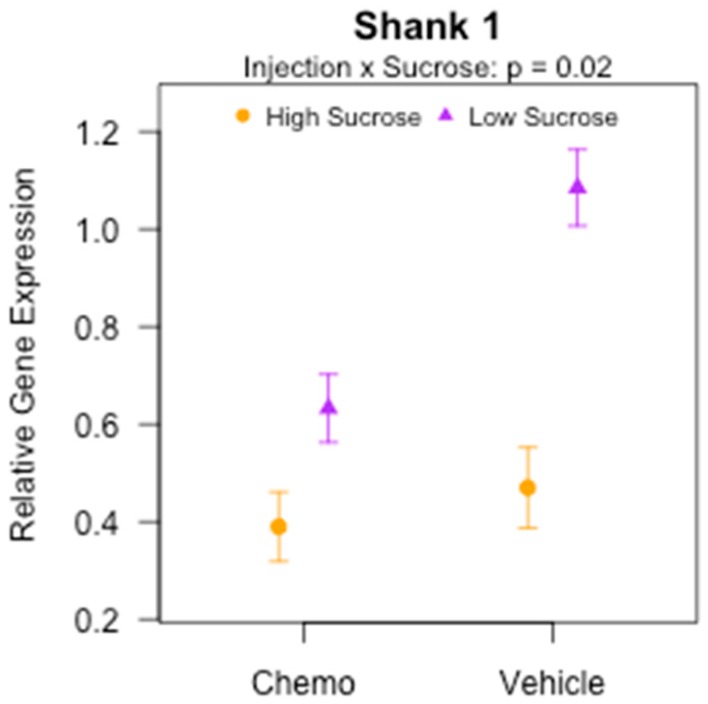
Significant injection by sucrose interaction effect on cortical gene expression of *Shank 1* in mice receiving various combinations of omega-3 and sucrose diets and two vehicle or chemotherapy injections (Study 2). mRNA measured by q-PCR. Data (means ± SEM, *n* = 11–14/group) were analyzed by 3-way ANOVA.

The postsynaptic density protein, *Shank 1*, varied significantly among experimental groups with a significant interaction between injection and sucrose (Figure 6; *p* < 0.0001). Among the low sucrose groups, the chemotherapy treated mice exhibited reduced *Shank 1* expression relative to the vehicle mice (*p* < 0.0001), but there was no effect of injection among the high sucrose groups (*p* > 0.05). *Shank 1* expression was also significantly lower for both chemotherapy and vehicle receiving mice (*p* < 0.05 and *p* < 0.0001, respectively) receiving high sucrose versus low sucrose diets. These data demonstrate the protective effect of the low sucrose diet on gene expression of *Shank 1*. Cortical *Shank 3* gene expression was also variable (Table 2; *p* < 0.05). It exhibited a main effect of injection (*p* < 0.05) and EPA + DHA (*p* < 0.01). Chemotherapy reduced cortical *Shank 3* expression relative to vehicle controls; similar to Study 1 (Figure 2), the 2% EPA + DHA diet increased the expression relative to the No EPA + DHA diet.

Table 2 shows hippocampal and cortical gene expression of *Tnf-α*, *Il-1β*, *Il-6* and *Nfκ-β, and Shank 3* quantified in study 2. The overall ANOVA F test was not significant for hippocampal *Tnf-α*, *Il-1β,* and *Il-6* (all *p* > 0.05). However, there was significant variation of *Nf-κβ* (*p* < 0.0001); there was an interaction between sucrose and injection (Figure 5A; *p* < 0.01) and between sucrose and EPA + DHA (Figure 5B; *p* < 0.0001) on gene expression of hippocampal *Nf-κβ*. 

### 3.9. Effects of Sucrose, Omega-3 Fatty Acids, and Chemotherapy on Oxidative Stress, Anti-Oxidant Defense and Lipid Peroxidation Markers in the Brain

In study 2, there were no differences in overall oxidative stress measured by Oxyblot assay, anti-oxidant defense measured by SOD activity assay, or lipid peroxidation in brain tissue assayed by 4-HNE levels (*p* > 0.05 for all).

## 4. Discussion

In this model of postmenopausal chemotherapy, two injections of doxorubicin-based chemotherapy induced significant neuroinflammation and reduced gene expression of dendritic anchoring proteins essential to synaptic function, when compared to vehicle treatment. Dietary enrichment with 2% kcal as EPA + DHA for five weeks resulted in higher brain DHA. Additionally, in chemotherapy-treated mice, brain DHA was higher with low versus high sucrose diets. Beneficial effects on gene expression of dendritic anchoring proteins in the cortex were seen with low sucrose and EPA + DHA in diets. EPA + DHA enriched diets resulted in lower levels of specific neuroinflammatory proteins in the cortex.

Our results support previous reports that doxorubicin-based chemotherapy causes significant neuroinflammation [11,12]. Further, we found that chemotherapy-induced inflammation varied depending on time since the last injection and the site of measurement (i.e., hippocampus, cortex or serum). Regardless of the time point of tissue collection (i.e., 4, 7, or 14 days post-chemotherapy), mice fed the 2% EPA + DHA diet had higher gene expression of cortical *Shank 3*, a member of a family of anchoring proteins that is essential to synaptic formation, development, and function [34]. Ergo, *Shank 3* expression contributes to brain plasticity. To our knowledge, this is the first report of a diet enriched with EPA + DHA altering gene expression of a SHANK family protein. The functional significance of this observation cannot be determined from the current study, but reduced *Shank 3* expression has been associated with autism-like behaviors, such as social deficits and stereotype [35,36].

Feeding sucrose during chemotherapy led to interesting effects on brain DHA. In chemotherapy-treated mice only, brain DHA was significantly higher with low sucrose versus high sucrose diets. As higher brain DHA has been associated with improved cognitive performance in animal models [37], this dietary effect warrants further study. The fact that sucrose only affected brain DHA in chemotherapy-treated mice suggests that high sucrose intake during doxorubicin-based chemotherapy may be interacting to reduce DHA incorporation or increase metabolism of DHA in the brain. Other researchers have reported that high sucrose diets resulted in elevated levels of brain arachidonic acid in rats and increased activity of phospholipase A2 (PLA2) enzymes, which are central to initiating metabolism of brain arachidonic acid and DHA by cleaving PUFAs from phospholipid membranes [23]. We did not see significant differences in brain arachidonic acid based on dietary sucrose alone, but we did find evidence that dietary sucrose in conjunction with chemotherapy alters levels of other n-6 PUFAs in the brain (e.g., lowers linoleic acid and raises arachidonic acid). Research suggests that doxorubicin may reduce activity of the DHA-preferring isoform of PLA2 (i.e., iPLA2) and increase arachidonic acid [38,39]. Inhibition of iPLA2 could lead to inability to repair damaged, oxidized lipids in membranes [40]. However, we did not find differences in the lipid peroxidation or oxidative stress markers that we measured. Future research is needed to clarify the effects of doxorubicin-based chemotherapy, n-3 PUFAs and sucrose on PUFA metabolites in the brain.

Chemotherapy reduced gene expression of *Shank 3* in the cortex, and increased protein levels of inflammatory markers IL-5 and IL-6 in the hippocampus, compared to vehicle treatment. In contrast, consumption of a 2% EPA + DHA diet had opposite effects: increasing *Shank 3* gene expression and reducing IL-6 protein levels in the cortex. Our study confirms previous reports that treatment with doxorubicin and cytoxan increases IL-6 in serum of patients [41] and brains of rodents [16]. However, both the chemotherapy and diet effects on *Shank 3* gene expression are novel; while measures of additional synaptic markers are warranted, our findings suggest that chemotherapy-induced deficits are partly mediated by detrimental changes in synaptic plasticity which can be attenuated by 2% EPA + DHA. Whether inflammation resulting from chemotherapy contributes to the observed decline in *Shank 3* is unknown, but a similar pattern of increased central nervous system IL-6 and decreased *Shank 3* has been reported following exposure to air pollution [42]. In addition, we found beneficial changes in the expression of the gene encoding for *Shank 1* and *Nfκ-β* in mice fed low versus high sucrose diets. High dietary sucrose has been linked to increased neuroinflammatory gene expression [43], but modulation of SHANK family genes by dietary sucrose has not been previously reported. Together, these data suggest that EPA + DHA and sucrose content of a diet may have effects on brain plasticity, which is particularly crucial for individuals undergoing chemotherapy treatment.

We observed differential effects of chemotherapy and 2% EPA + DHA on neuroinflammation in different regions of the brain. This could be related to the more “alert” or responsive phenotype of the immune cells in the hippocampus compared to the cortex [44]. Indeed, we did find that chemotherapy induced significant inflammation in the hippocampus but not the cortex 10 days after two injections. Additionally, EPA + DHA reduced inflammation in the cortex, but not the hippocampus at this time point. It is possible that region-specific differences in concentration of brain DHA also may have contributed to differential effects, as research suggests that there is a greater proportion of DHA in the cortex versus hippocampus of C57BL/6 mice [45]. We were unable to investigate this possibility because we measured fatty acids using whole brain tissue and not distinct brain regions.

We found no significant differences in oxidative stress markers among diet or chemotherapy-treated groups. In contrast, a recent report suggests that supplementation of n-3 PUFAs alleviates doxorubicin-induced oxidative stress in male rats [16]. Our diverging results could be related to differences in the model used, delivery of n-3 PUFAs (i.e., diet enrichment in our study versus gavage by Wu and colleagues) or the dose and delivery of chemotherapy. Additionally, our inability to detect significant differences in oxidative stress could be partially related to the relatively high alpha linolenic acid (ALA) content of our No EPA + DHA control diet. While not considered to be as anti-inflammatory as the longer chain, marine-derived n-3 PUFAs, ALA does possess some anti-inflammatory properties [46] and is more efficiently converted to DHA by rodents than humans [47]. Recently, researchers reported that a low sucrose diet enriched with ALA and fiber countered the effects of high sucrose/high fat diet-induced obesity on spatial and short-term memory in rats, and improved oxidative stress markers [47]. Future research should examine the effects of a diet with various amounts of ALA and EPA + DHA on chemotherapy-induced neuroinflammation and oxidative stress. Another promising area for future research centers on resolution of inflammation. Specialized pro-resolving mediators (SPMs) of n-3 PUFAs, such as resolvins and protectins, have been detected in the hippocampus of mouse models of lipopolysaccharide-induced inflammation and humans with Alzheimer’s disease [48,49]. Future studies of SPMs in the context of chemotherapy could add to our knowledge of mechanisms underlying nutritional modulation of neuroinflammation.

Strengths of this study include the measurement of central and peripheral inflammation at several time points post-chemotherapy. In addition, the dose of EPA + DHA was comparable, as percentage of energy, to high dose supplementation studies in breast cancer survivors and in women at high risk for breast cancer [50,51], and similar to doses shown to reduce neuroinflammation [52,53] and tumors [54] in mice. The sucrose doses were also designed to test effects across a range of intakes physiologically relevant to humans [55]. Additionally, to make this research more translatable to the human condition, the diets were formulated to avoid inducing an n-3 PUFA deficiency; the percentage of energy provided by ALA in the control diets was approximately twice the median intake of U.S. adults [56]. However, this is also a limitation because we did not include a diet low in total n-3 PUFAs, which could have reduce our ability to detect differences between groups. Also, we were unable to separate the effects of EPA from DHA in our studies. Finally, the use of relatively young healthy mice that had not had tumors may have altered the physiological response to chemotherapy. 

In conclusion, this research provided the first evidence that both chemotherapy and diet influence of *Shank* gene expression, which may have implications for brain plasticity and function. Both low sucrose and EPA + DHA in the diet may attenuate neuroinflammation induced by doxorubicin-based chemotherapy and protect proteins important in synaptic formation and function in specific brain regions. Further research is needed to examine effects of dietary modification on chemotherapy-induced cognitive deficits.

## Figures and Tables

**Table 1:** Protein concentrations of inflammatory and synaptic markers in serum, the hippocampus, and the cortex in mice receiving various combinations of omega-3 and sucrose diets and two injections of chemotherapy or vehicle (Study 2).

	Chemotherapy								Vehicle							
	2% EPA + DHA		No EPA + DHA		2% EPA + DHA		No EPA + DHA		2% EPA + DHA		No EPA + DHA		2% EPA + DHA		No EPA + DHA	
	Low Sucrose		Low Sucrose		High Sucrose		High Sucrose		Low Sucrose		Low Sucrose		High Sucrose		High Sucrose	
**Serum**																
IFN-γ ^#^	0.78	(0.33)	0.73	0.44	0.62	0.18	0.60	0.27	0.44	0.16	0.43	0.24	0.54	0.26	0.41	0.30
IL-10	9.0	(4.5)	7.7	(1.7)	8.1	(2.8)	9.0	(2.7)	7.7	(1.2)	9.0	(3.5)	7.7	(1.8)	7.3	(3.2)
IL-1b ^^^	1.6	(0.56)	0.91	(0.62)	1.3	(0.50)	1.6	(0.52)	0.48	(0.24)	0.36	(0.14)	0.67	(0.21)	0.46	(0.33)
IL-2	1.3	(1.6)	1.5	(0.88)	1.1	(0.55)	1.0	(0.38)	0.60	(0.38)	0.87	(0.50)	0.52	(0.24)	0.73	(0.26)
IL-5 ^#^	9.1	(6.1)	9.5	(6.9)	8.5	(4.9)	10.1	(4.4)	2.7	(1.1)	2.7	(1.4)	1.9	(0.57)	2.9	(1.2)
IL-6 ^#^	16.7	(7.4)	21.1	(16)	16.7	(7.1)	19.2	(5.5)	4.0	(2.6)	4.5	(2.9)	3.6	(2.0)	2.1	(2.0)
KC/GRO	73.6	(26)	85.6	(34)	84.6	(20)	90.5	(21)	94.0	(19)	94.1	(23)	100.8	(12)	95.6	(16)
TNF-a ^#^	10.3	(2.2)	11.8	(5.5)	11.4	(3.6)	11.1	(2.2)	7.2	(2.4)	7.5	(2.5)	8.5	(2.1)	9.4	(2.5)
**Hippocampus**																
IL-10	1.5	(0.55)	1.5	(0.59)	0.98	(0.51)	1.3	(0.75)	0.86	(0.53)	1.09	(0.76)	1.1	(0.57)	0.74	(0.85)
IL-5 ^#,Ω^	1.2	(0.37)	1.2	(0.43)	1.1	(0.39)	0.98	(0.32)	1.0	(0.33)	0.74	(0.18)	1.1	(0.42)	0.91	(0.23)
IL-6 ^#,Ω^	19.0	(4.2)	18.7	(5)	18.5	(5.5)	16.1	(3.8)	17.4	(4.1)	12.9	(2.5)	17.2	(5.1)	15.1	(3.0)
**Cortex**																
IFN-γ	0.088	(0.052)	0.161	(0.086)	0.100	(0.094)	0.108	(0.061)	0.132	(0.099)	0.088	(0.049)	0.115	(0.068)	0.073	(0.031)
IL-10	1.3	(0.67)	1.7	(0.88)	1.1	(0.72)	1.4	(0.45)	1.2	(0.35)	1.2	(0.55)	1.3	(0.65)	1.1	(0.58)
IL-12	13	(7.0)	18	(9.9)	12	(4.9)	15	(8.0)	13	(9.2)	15	(4.0)	14	(4.9)	15	(3.5)
IL-1b	0.21	(0.13)	0.28	(0.15)	0.43	(0.55)	0.23	(0.07)	0.28	(0.35)	0.29	(0.13)	0.26	(0.12)	0.40	(0.25)
IL-2	0.83	(0.33)	0.98	(0.28)	1.22	(1.49)	0.85	(0.26)	1.05	(0.49)	1.12	(0.41)	0.87	(0.30)	0.84	(0.18)
IL-4	0.31	(0.18)	0.55	(0.20)	0.43	(0.16)	0.44	(0.19)	0.37	(0.22)	0.42	(0.17)	0.46	(0.14)	0.51	(0.15)
IL-5 ^Ω^	1.2	(0.23)	1.4	(0.27)	1.1	(0.40)	1.3	(0.28)	1.1	(0.34)	1.4	(0.23)	1.3	(0.30)	1.5	(0.29)
IL-6 ^Ω^	17	(3.3)	20	(3.7)	17	(5.6)	18	(4.0)	16	(4.1)	20	(3.0)	18	(3.3)	21	(4.1)
KC/GRO ^Ω^	0.39	(0.28)	0.80	(0.38)	0.41	(0.25)	0.57	(0.26)	0.46	(0.34)	0.52	(0.25)	0.40	(0.19)	0.43	(0.18)
TNF-a	0.32	(0.17)	0.45	(0.22)	0.26	(0.14)	0.28	(0.09)	0.31	(0.22)	0.35	(0.14)	0.31	(0.18)	0.37	(0.12)

Data are shown as mean (SD), pg/mL; **^#^** Significant main effect of injection; *p* < 0.01; **^^^** Significant 3-way interaction effect of injection by sucrose by EPA + DHA; *p* < 0.01 (See Figure 4); **^Ω^** Significant main effect of EPA + DHA; *p* < 0.05.

**Table 2:** Gene expression of inflammatory and synaptic markers in the hippocampus and the cortex in mice receiving various combinations of omega-3 and sucrose diets and two injections of chemotherapy or vehicle (Study 2).

	Chemo								Vehicle							
	2% EPA + DHA		No EPA + DHA		2% EPA + DHA		No EPA + DHA		2% EPA + DHA		No EPA + DHA		2% EPA + DHA		No EPA + DHA	
	Low Sucrose		Low Sucrose		High Sucrose		High Sucrose		Low Sucrose		Low Sucrose		High Sucrose		High Sucrose	
**Hippocampus**																
TNF-a	2.0	(1.4)	1.3	(0.82)	0.82	(0.6)	2.0	(1.7)	1.2	(1.3)	0.65	(0.51)	1.3	(1.3)	1.2	(0.93)
IL-1b	2.4	(1.9)	1.0	(1.0)	0.66	(0.4)	2.5	(3.1)	1.1	(0.85)	0.4	(0.31)	2.1	(2.9)	1.8	(1.8)
IL-6	1.0	(0.53)	1.12	(0.45)	1.0	(0.47)	1.4	(0.90)	1.22	(0.83)	1.11	(0.60)	0.99	(0.47)	0.99	(0.36)
Nfk-b ^π,ω^	1.1	(0.31)	0.57	(0.14)	0.82	(0.18)	0.95	(0.25)	0.98	(0.37)	0.41	(0.10)	1.05	(0.31)	1.02	(0.21)
Shank 3	1.0	(0.72)	1.0	0.72	0.73	(0.47)	0.84	(0.46)	1.2	(0.66)	1.1	(0.58)	1.2	(0.76)	1.2	(0.65)
**Cortex**																
TNF-a	1.1	(0.49)	0.66	(0.36)	1.2	(0.49)	0.73	(0.33)	1.0	(0.58)	0.88	(0.34)	1.1	(0.56)	1.1	(0.42)
IL-1b	1.3	(0.66)	0.89	(0.30)	1.4	(0.49)	1.3	(0.77)	1.1	(0.55)	1.1	(0.39)	1.4	(0.85)	1.1	(0.36)
IL-6	1.1	(0.59)	0.82	(0.43)	1.4	(0.66)	1.1	(0.48)	1.2	(0.69)	1.1	(0.28)	1.0	(0.36)	1.1	(0.32)
Nfk-b	0.93	(0.25)	0.91	(0.30)	1.1	(0.27)	0.95	(0.16)	1.0	(0.26)	1.0	(0.32)	1.0	(0.20)	1.0	(0.19)
Shank 3 ^#,Ω^	1.0	(0.20)	0.82	(0.20)	1.0	(0.24)	0.87	(0.12)	1.1	(0.19)	1.0	(0.20)	1.0	(0.20)	0.98	(0.16)
Shank 1 ^π^	0.71	(0.27)	0.55	(0.20)	0.49	(0.17)	0.29	(0.15)	0.98	(0.55)	1.2	(0.61)	0.61	(0.36)	0.33	(0.16)

Data are shown as mean (SD) of gene expression relative to endogenous control, eukaryotic 18S; **^π^** Significant 2-way interaction effect of injection by sucrose; *p* < 0.05 (See Figure 5A and Figure 6); **^ω^** Significant 2-way interaction effect of EPA + DHA by sucrose; *p* < 0.0001; (See Figure 5B); **^#^** Significant main effect of injection; *p* < 0.05; **^Ω^** Significant main effect of EPA + DHA; *p* < 0.01.

## Supplementary Materials

The following are available online at http://www.mdpi.com/2072-6643/10/12/2004/s1, Table S1: Mouse Diet Formulation, Table S2: Fatty acid composition of mouse diets, Table S3: TaqMan primer/probes used in qPCR, Table S4: Cumulative food intake and body weight over time by diet and injection groups in mice receiving 2% EPA + DHA or No EPA + DHA diets and two injections of vehicle or chemotherapy (Study 1), Table S5: Cumulative food intake and body weight over time in mice receiving various combinations of omega-3 and sucrose diets and two injections of chemotherapy (Study 2), Table S6: Brain fatty acids in mice fed varying n-3 PUFA and sucrose enriched diets for 5 weeks while undergoing two chemotherapy or vehicle injections.
