# Supplementary material for: Low Sucrose, Omega-3 Enriched Diet Has Region-Specific Effects on Neuroinflammation and Synaptic Function Markers in a Mouse Model of Doxorubicin-Based Chemotherapy
Source: Nutrients. 2018 Dec 18;10(12):2004. doi: 10.3390/nu10122004 (PMC6316589; doi:10.3390/nu10122004)
Supplement: Supplementary file 1 [file nutrients-10-02004-s001.pdf]

Supplementary Materials

Table S1. Mouse Diet Formulations [a].

	NO EPA+DHA/ LOW SUCROSE	NO EPA+DHA/ HIGH SUCROSE	2% EPA+DHA/ LOW SUCROSE	2% EPA+DHA/ HIGH SUCROSE
**Ingredient**	gm/kg	gm/kg	gm/kg	gm/kg
Casein	232.5	232.5	205	205
DL-Methionine	3	3	3	3
Corn Starch	450	100	450	100
Sucrose	100	500	100	500
Maltodextrin10	100	50	100	50
Cellulose	50	50	50	50
Soybean Oil	90	90	41	41
MEG-3, 30% Powder	0	0	81	81
Mineral mix	35	35	35	35
Vitamin mix	10	10	10	10
Choline	2	2	2	2
Protein (gm%)	19	19	19	19
Carbohydrate (gm%)	61	61	61	61
Fat (gm%)	8	8	8	8
Protein (kcal%)	19	19	19	19
Carbohydrate (kcal%)	61	61	61	61
Fat (kcal%)	19	19	19	19

[a] Diets prepared by Research Diets, Inc.

Table S2. Fatty acid composition of mouse diets [a].

	Diets							
**Dietary Fatty Acid**	**NO EPA+DHA/ LOW SUCROSE**		**NO EPA+DHA/ HIGH SUCROSE**		**2% EPA+DHA/ LOW SUCROSE**		**2% EPA+DHA/ HIGH SUCROSE**	
	Mean %	***SD***	***Mean %***	***SD***	***Mean %***	***SD***	***Mean %***	***SD***
**C14:0**	0.3127	0.0056	0.3465	0.0183	4.0342	0.0655	4.0396	0.1155
**C16:0**	10.8571	0.0044	10.8156	0.0162	15.6643	0.0515	15.6578	0.1455
**C16:1n7**	0.1246	0.0102	0.1256	0.0121	4.7284	0.0682	4.7533	0.1353
**C16:2n4**	ND [b]		ND		0.6467	0.0136	0.6746	0.0129
**C16:3n4**	0.0585	0.0033	0.0580	0.0018	0.7979	0.0132	0.7923	0.0207
**C18:0**	3.7595	0.0075	3.7911	0.0128	3.7329	0.0262	3.8003	0.0131
**C18:1n9**	19.9944	0.0661	19.9874	0.0771	14.3479	0.0526	14.5019	0.1299
**C18:1n7**	1.4386	0.0437	1.4651	0.0507	2.1407	0.0437	2.1796	0.0354
**C18:2n6**	55.7595	0.0116	55.7239	0.0613	28.0789	0.4165	27.4273	0.8709
**C18:3n6**	ND		ND		0.1622	0.0088	0.1744	0.0152
**C18:3n3**	7.1302	0.0301	7.1640	0.0585	4.0564	0.0457	3.9617	0.0978
**C18:4n3**	ND		ND		2.1081	0.0256	2.1021	0.0458
**C20:0**	0.2380	0.0153	0.2492	0.0068	0.4335	0.0129	0.4489	0.0220
**C20:1n9**	0.1587	0.0057	0.1676	0.0039	0.8631	0.0403	0.9112	0.0393
**C20:2n6**	0.0424	0.0044	ND		0.1139	0.0040	0.1141	0.0067
**C20:3n6**	ND		ND		0.0954	0.0057	0.0990	0.0071
**C20:4n6**	ND		ND		0.4834	0.0083	0.4938	0.0109
**C20:4n3**	ND		ND		0.5428	0.0136	0.5548	0.0225
**C20:5n3**	ND		ND		9.2107	0.1225	9.3503	0.2936
**C22:4n6**	0.0989	0.0152	0.0988	0.0067	0.1218	0.0623	0.1128	0.0966
**C22:5n6**	ND		ND		0.1417	0.0131	0.1481	0.0094
**C22:5n3**	0.0740	0.0256	0.0602	0.0035	0.8908	0.0689	0.9321	0.1051
**C22:6n3**	ND		ND		6.6043	0.1001	6.7700	0.2156
**Total n-3**	7.2042	0.0501	7.2041	0.0238	23.4131	0.3142	23.6710	0.5725
**Total n-6**	55.8537	0.0765	55.7897	0.0145	29.1974	0.4603	28.5695	0.9279
**n-3/n-6**	0.1290	0.0011	0.1291	0.0004	0.8021	0.0231	0.8296	0.0470
**n-6/n-3**	7.7533	0.0642	7.7442	0.0254	1.2474	0.0364	1.2080	0.0685

[a] Values are averages of triplicate analysis by gas chromatography reported as percentage of total fatty acids. [b] ND=Not detectable.

Table S3. TaqMan primer/probes used in qPCR.

**Gene**	**Assay ID**
Tnfa	Mm00443260_g1
Il1b	Mm00434228_m1
Il6	Mm00446190_m1
Nfkb1	Mm00476361_m1
Shank3	Mm00683065_m1
Shank1	Mm01206737_m1
Dlg4 (PSD 95)	Mm00492193_m1

Table S4. Cumulative food intake and body weight over time by diet and injection groups in mice receiving 2% EPA+DHA or No EPA+DHA diets and two injections of vehicle or chemotherapy (Study 1).

	Chemo				Vehicle			
	2% EPA+DHA		No EPA+DHA		2% EPA+DHA		No EPA+DHA	
Food Consumption								
4 days post-injection	97.8	(7.5)	102.6	(8.9)	100.9	(8.9)	103.7	(6.0)
7 days post-injection	96.6	(11)	104.0	(8.2)	98.2	(10)	106.9	(8.1)
14 days post-injection	102.9	(4.6)	109.5	(7.1)	103.1	(14)	108.5	(4.5)
Body Weight								
Experiment day 1	18.9	(0.91)	19.3	(1.3)	18.7	(1.2)	19.0	(1.2)
Experiment day 14	20.9	(1.5)	20.8	(1.5)	20.9	(1.7)	20.5	(2.4)
Experiment day 28	20.2	(1.3)	20.0	(1.4)	21.2	(2.0)	20.8	(1.3)

Data are shown as mean (SD), grams

Table S5: Cumulative food intake and body weight over time in mice receiving various combinations of omega-3 and sucrose diets and two injections of chemotherapy (Study 2)

	Chemo								Vehicle							
	2% EPA+DHA Low sucrose		No EPA+DHA Low sucrose		2% EPA+DHA High sucrose		No EPA+DHA High sucrose		2% EPA+DHA Low sucrose		No EPA+DHA Low sucrose		2% EPA+DHA High sucrose		No EPA+DHA High sucrose	
Food Consumption	87.8	(4.8)	94.7	(4.5)	94.6	(11.3)	97.1	(5.9)	92.5	(10.5)	97.3	(6.6)	98.8	(5.6)	101.3	(7.2)
Body Weight																
Experiment day 1	19.1	(1.6)	19.7	(2.1)	20.4	(2.1)	19.8	(1.9)	20.1	(2.0)	19.0	(2.1)	20.5	(1.8)	20.1	(1.5)
Experiment day 7	20.1	(1.6)	20.7	(1.5)	21.8	(1.8)	21.0	(1.8)	20.6	(2.5)	20.3	(1.8)	21.9	(1.2)	20.5	(1.3)
Experiment day 21	19.3	(1.1)	20.0	(2.4)	21.2	(1.8)	20.4	(1.6)	21.8	(1.2)	20.5	(2.2)	22.5	(1.4)	21.3	(2.0)

Data are shown as mean (SD)

Table S6: Brain fatty acids in mice fed varying n-3 FA and sucrose enriched diets for 5 weeks while undergoing two chemotherapy or vehicle injections [a]

	Diet Groups [b]								
**Brain Fatty Acid**	**NO EPA+DHA/ LOW SUCROSE**		**NO EPA+DHA/ HIGH SUCROSE**		**2% EPA+DHA/ LOW SUCROSE**		**2% EPA+DHA/ HIGH SUCROSE**		
	Mean %	***SD***	***Mean %***	***SD***	***Mean %***	***SD***	***Mean %***	***SD***	***p*** [c]
**C14:0**	0.1391	0.0136	0.1381	0.0118	0.1563	0.0158	0.1478	0.0090	**<.0001**
**C16:0**	20.1449	2.0744	19.9560	2.1619	20.8191	2.0066	19.9090	2.0481	0.30
**C16:1n7**	0.4200	0.0441	0.4207	0.0441	0.4986	0.0453	0.4819	0.0422	**<.0001**
**C18:0**	21.4593	1.1054	21.2381	1.1453	21.7732	1.1227	21.1665	1.2914	0.38
**C18:1n7**	17.3091	2.3497	17.5575	2.4748	17.3809	2.5730	18.5290	2.7236	0.14
**C18:1n9**	3.6183	0.5099	3.6769	0.5413	3.3624	0.5143	3.5515	0.4935	0.40
**C18:2n6**	0.7304	0.1188	0.6755	0.0928	0.5346	0.0962	0.4802	0.0939	**<.0001**
**C18:3n6**	0.0514	0.0106	0.0516	0.0125	0.0485	0.0097	0.0518	0.0137	0.49
**C20:0**	0.4806	0.2066	0.4974	0.2138	0.4062	0.1785	0.4907	0.2069	0.31
**C20:1n9**	2.1975	1.1401	2.2741	1.1738	1.7018	1.0857	2.2318	1.1599	0.25
**C20:2n6**	0.1842	0.0721	0.1783	0.0691	0.1112	0.0408	0.1187	0.0400	**<.0001**
**C20:3n6**	0.4556	0.0483	0.4633	0.0477	0.4436	0.0353	0.4639	0.0344	0.12
**C20:4n6**	8.9350	1.4886	9.0276	1.5694	7.9746	1.5138	7.6110	1.4950	**0.02**
**C20:5n3**	0.0772	0.0738	0.0638	0.0553	0.2836	0.0625	0.2732	0.0672	**<.0001**
**C22:0**	0.5722	0.2322	0.5889	0.2497	0.4810	0.2030	0.6015	0.2519	0.34
**C22:4n6**	2.4320	0.3046	2.5176	0.2846	1.9197	0.2835	1.9721	0.2291	**<.0001**
**C22:5n3**	0.1924	0.0695	0.2364	0.1287	0.2014	0.0945	0.1773	0.0843	**<.0001**
**C22:5n6**	0.1837	0.0878	0.1532	0.0727	0.4430	0.0901	0.4515	0.0704	0.52
**C22:6n3**	19.2151	0.9643	19.0578	0.6641	20.4779	0.8827	20.0303	0.8260	**<.0001**
**C24:0**	0.9148	0.4048	0.9426	0.4832	0.7514	0.3494	0.9930	0.4332	0.33
**AA/DHA**	0.4660	0.0819	0.4739	0.0824	0.3894	0.0773	0.3796	0.0713	**<.0001**

[a] Values obtained by gas chromatography, reported as percentage of total fatty acids in brain, averaging across injection treatment groups (chemotherapy and vehicle)
[b] n=23-25 per diet group
[c] P-value obtained from overall ANOVA F-test
